# Evaluation of European severe acute respiratory infection (SARI) surveillance, 27 European countries, 2022/23

**DOI:** 10.2807/1560-7917.ES.2025.30.20.2400655

**Published:** 2025-05-22

**Authors:** Diogo FP Marques, Dory Kovacs, Miguel-Angel Sanchez-Ruiz, Ana Paula Rodrigues, Ausenda Machado, Clara Mazagatos, Susana Monge, Lisa Domegan, Joan O’Donnell, Mariette Hooiveld, Hanne-Dorthe Emborg, Baltazar Nunes, Carlos Carvalho, Angela MC Rose

**Affiliations:** 1 Epiconcept, Paris, France; 2 NOVA National School of Public Health, Public Health Research Centre, Comprehensive Health Research Center, NOVA University Lisbon, Lisbon, Portugal; 3 Instituto Nacional de Saúde Doutor Ricardo Jorge, Lisbon, Portugal; 4 Communicable Diseases Department, National Centre of Epidemiology, Institute of Health Carlos III, Madrid, Spain; 5 CIBERESP, Madrid, Spain; 6 CIBERINFEC, Madrid, Spain; 7 HSE Health Protection Surveillance Centre, Dublin, Ireland; 8 Nivel, Utrecht, the Netherlands; 9 Statens Serum Institut, Copenhagen, Denmark; 10 European Centre for Disease Prevention and Control (ECDC), Stockholm, Sweden

**Keywords:** surveillance, severe acute respiratory infection (SARI), evaluation, SARS-CoV-2, influenza, respiratory syncytial virus (RSV)

## Abstract

**Background:**

Between 2020 and 2023, ECDC has supported 21 of 30 EU/EEA and six Western Balkan countries by enhancing severe acute respiratory infection (SARI) surveillance to monitor trends, detect unexpected events, evaluate public health interventions, identify risk factors and support vaccine effectiveness studies. Using diverse strategies, countries have implemented SARI surveillance and reported data at national/European levels.

**Aim:**

We evaluated European-level SARI surveillance and provided recommendations to achieve objectives and improve key attribute performance.

**Methods:**

We analysed 2022/23 surveillance data for completeness. We administered a questionnaire, targeting country-level representatives, to evaluate surveillance attributes (meeting objectives, usefulness, acceptability, timeliness, representativeness) and identify strengths, weaknesses, opportunities and threats.

**Results:**

Thirteen countries (13/27) reported data at European level. Data showed good overall completeness but varied across countries and some variables need improvement (vaccination, sequencing). The questionnaire was completed by all 27 countries. Most countries (23/27) reported that the system effectively monitored trends and considered it useful and acceptable (25/27), but only 16 found it timely and 14 representative. Challenges included insufficient case-based data, data linkage issues and insufficient data completeness. Slow/inefficient manual data extraction affected timeliness, while insufficient geographical coverage affected representativeness. Multi-pathogen surveillance was identified as the main strength, heterogeneity of systems the main weakness, improvements of hospital information systems the main opportunity, and lack of sustainable funding the main threat.

**Conclusions:**

SARI surveillance was perceived as effective in monitoring trends, useful and acceptable. To achieve additional objectives and enhance timeliness and representativeness, we recommend improving data completeness, digitalisation/automation and geographical coverage.

**Key public health message**

**What did you want to address in this study and why?**

Between 2020 and 2023, the European Centre for Disease Prevention and Control has supported 27 countries (21/30 EU/EEA and 6 Western Balkans countries) in strengthening their severe acute respiratory infection (SARI) surveillance systems. We evaluated the performance of European SARI surveillance (2022/23) and provided recommendations to help achieve its objectives and improve performance on key surveillance attributes.

**What have we learnt from this study?**

European SARI surveillance was useful, acceptable and able to meet its main objective of monitoring trends of severe respiratory disease. However, improvements are needed in terms of timeliness, representativeness and data completeness to allow the system to fulfil all of its objectives.

**What are the implications of your findings for public health?**

The study results provide an opportunity to improve key attributes such as timeliness, representativeness and data completeness of SARI surveillance. Such improvements would allow the system to identify risk factors that may predispose patients to severe outcomes, monitor the effects of public health interventions including vaccination and further contribute to pandemic preparedness and response.

## Introduction

Robust integrated syndromic surveillance of severe acute respiratory infections (SARI) is key for early detection and monitoring of pandemics, especially when diagnostic capacity is limited [1,2]. Syndromic surveillance also allows greater understanding of the overall burden of respiratory infections, whereas surveillance of laboratory-confirmed cases often underestimates the burden [3].

During the 2009 influenza A(H1N1)pdm09 pandemic, monitoring the incidence of severe respiratory disease and identification of its aetiology was challenging because of the lack of hospital-based surveillance systems [4]. In subsequent years, the World Health Organization (WHO) recommended establishing national SARI surveillance to monitor severe disease, enable evaluation of interventions and improve preparedness and emergency response [4]. However, most EU/EEA countries with severe respiratory disease surveillance systems focused on influenza-positive patients admitted to intensive care unit (ICU) [5,6].

In 2020, the COVID-19 pandemic exposed the persisting weaknesses of surveillance [7] and prompted European Centre for Disease Prevention and Control (ECDC) and WHO to renew their recommendations for countries to implement integrated hospital surveillance of respiratory viral infections [8]. In response, various initiatives were launched to strengthen SARI surveillance, including the ECDC-funded projects: Vaccine Effectiveness, Burden and Impact Studies (VEBIS) and ‘Design and implementation of multinational surveillance systems using routinely collected electronic health records in EU/EEA’ – Surveillance from Electronic Health Data (SUREHD) project [9-11], which aim to strengthen SARI surveillance systems in 27 countries (21/30 European Union/European Economic Area (EU/EEA) countries and six from the Western Balkans).

The European SARI surveillance comprises national surveillance systems that ECDC coordinates at supranational level. Its objectives are to (i) monitor trends of SARI and their impact on hospitalisations and in-hospital mortality; (ii) ensure the early detection and response to unusual and unexpected events caused by common or emerging respiratory pathogens; (iii) assess the impact of public health interventions, including vaccination, on respiratory infections and inform disease preparedness, prevention and control; (iv) identify risk factors for SARI and death; and (v) contribute to pathogen-specific SARI vaccine effectiveness monitoring. The system uses syndromic case definitions to identify patients admitted to emergency or respiratory wards, based on symptoms (e.g. WHO SARI case definition [12]). In absence of symptoms, diagnostic codes indicative of SARI may be used as a proxy of SARI [13]. Cases are identified throughout the year and routinely tested using multiplex PCR assays to detect selected respiratory pathogens including SARS-CoV-2, influenza and respiratory syncytial virus (RSV) [8]. Surveillance questionnaires and/or data linkage with existing databases are used to obtain information about patient age, sex, laboratory tests and results, key dates (hospital admission, laboratory sample, ICU admission, death) and outcome (discharge or death). Each week, data are consolidated at the national level and submitted to ECDC [13]. Key surveillance indicators are then presented on a weekly basis through the European Respiratory Virus Surveillance Summary (ERVISS) dashboard [14].

Several European countries have made substantial efforts to establish SARI surveillance systems, despite facing challenges in collecting the syndromic data needed to meet surveillance objectives [5,15-20]. Periodic evaluation of surveillance systems is recommended to ensure they meet their objectives, produce good-quality data, and remain efficient and useful to public health [21]. Previous evaluations at the EU level have been performed, but they focused on the evaluation of the surveillance of severe influenza [6,22] or COVID-19 surveillance [23] rather than SARI. In light of recent developments during the COVID-19 pandemic and the efforts made by countries and ECDC to strengthen SARI surveillance, we evaluated European-level SARI surveillance during the 2022/23 epidemiological year and provided recommendations to help achieve its objectives and improve performance on key surveillance attributes.

## Methods

### Surveillance evaluation

The evaluation team included members of the VEBIS and SUREHD project consortia, including representatives from national public health institutes and Epiconcept (project coordinator), as well as colleagues from ECDC.

This evaluation focused on the 27 European countries participating in ECDC-funded projects to strengthen SARI surveillance: Albania, Austria, Belgium, Bosnia and Herzegovina, Bulgaria, Croatia, Cyprus, Czechia, Denmark, Estonia, France, Germany, Iceland, Ireland, Italy, Kosovo^§^, Lithuania, Luxembourg, Malta, Montenegro, Netherlands, North Macedonia, Norway, Portugal, Romania, Serbia and Spain. We reviewed their country-specific surveillance protocols and project annual reports between February and May 2024 to describe their SARI surveillance system characteristics. Nine EU/EEA countries were not included as they were not active participants of the ECDC-funded projects at the time: Finland, Greece, Hungary, Latvia, Liechtenstein, Poland, Slovakia, Slovenia and Sweden.

We evaluated European SARI surveillance using methodologies proposed by the United States Centers for Disease Control and Prevention [24] and ECDC [21]. In June 2023, we held an online workshop to develop the questionnaire that was used to assess five surveillance attributes (whether the surveillance is meeting its objectives, usefulness, acceptability, timeliness and representativeness) and to identify the main strengths and weaknesses of, and opportunities and threats (SWOTs) to European SARI surveillance. We assessed data completeness through analysis of surveillance data from the 2022/23 epidemiological year. An overview of the attributes evaluated, their definitions and methods used is shown in Table 1.

**Table 1:** Overview of surveillance attributes and SWOTs, their definitions and data collection methods used, 27 European countries, 2022/23

Evaluation parameter	Definition	Online questionnaire	Surveillance data analysis
Surveillance attribute			
Data completeness	Completeness of weekly reporting: the proportion of weeks with data reported to ECDC Internal completeness: the proportion of complete records in a dataset, meaning they contain no missing (blank) values or values coded as ‘unknown’	NA	X
Meeting surveillance objectives	The extent to which the surveillance system has met its objectives	X	NA
Usefulness	The ability of the surveillance system to provide data for public health action	X	
Acceptability	The willingness to participate in the surveillance system and to provide accurate, consistent, complete and timely data	X	
Timeliness	The time interval between steps of the data flow in a surveillance system	X	
Representativeness	The ability to accurately describe the occurrence of cases in the population by place and age	X	
SWOT			
Strengths	Internal factors, within organisation control, that can enable, create and sustain success	X	NA
Weaknesses	Internal factors, within organisation control, that create vulnerability or prevent achieving objectives	X	
Opportunities	External factors, uncontrollable, that can make surveillance more efficient	X	
Threats	External factors, uncontrollable, that can negatively impact surveillance	X	

ECDC: European Centre for Disease Prevention and Control; NA: not applicable; SARI: severe acute respiratory infection; SWOT: strengths, weaknesses, opportunities and threats.

### Surveillance data analysis

We extracted data on SARI hospitalisations from the European Surveillance System (TESSy), a central data platform hosted by ECDC [25], from 3 October 2022 (week 40) to 1 October 2023 (week 39). Data extraction on 2 November 2023 provided a 4-week period following the end of the surveillance window to accommodate retrospective improvements to the data. We selected this timeframe to exclude earlier epidemiological years (when systems were transitioning from COVID-19 reporting to SARI surveillance) and to avoid variability introduced by changes to TESSy reporting protocols in October 2023, both of which could have affected data completeness and comparability.

The extract included three TESSy record types (TESSy reporting protocol v3.8) [13]: (i) INFLSARIAGGR.v3 for aggregated data, where we assessed SARI hospitalisations, ICU admissions, SARI deaths, all-cause admissions, catchment population, numbers of tests conducted and positive results for SARS-CoV-2 and influenza; (ii) SARISURV.v2 for case-based reporting, where we evaluated age, sex, dates (symptom onset, hospital admission, ICU admission, outcome, specimen collection, COVID-19 vaccination), ICU admission, outcome, respiratory support, symptoms, pre-existing conditions, laboratory results, vaccination and antiviral treatment; and (iii) SARISURVDENOM.v1 for reporting of denominators complementing the case-based data, where we examined catchment population and all-cause admissions.

We assessed two data completeness indicators at the country and European levels: the completeness of weekly reporting and internal data completeness [21]. The completeness of weekly reporting was calculated by dividing the number of weeks with data reported by the total number of weeks with data expected in the year. Internal data completeness was determined by dividing the number of complete records (not missing or unknown), by the total number of records (number of weeks with data reported for aggregated data, e.g. 33 of 52 weeks, or number of eligible records for case-based data, e.g. number of pregnancies among females). Internal data completeness was only estimated for weeks with data reported and for the variables reported at least once during the year.

### Online workshop and questionnaire

The target audience for the workshop and questionnaire included the surveillance system representatives from the 27 countries, as nominated by their national focal points for surveillance. Details on the number of representatives, workshop attendance and questionnaire responses per country are provided in Supplementary Table S1. We did not set a fixed number of representatives per country; participation was left at country’s discretion. Six members of the project consortia also represented their national public health institutes, making them both part of the evaluation team and the target audience, representing three of the 27 countries. The workshop was also attended by members of the evaluation team, as well as ECDC and WHO colleagues, who contributed to discussions and provided additional context. 

The workshop took place via the Zoom online meeting platform (https://zoom.us). Participants reflected on the attributes under evaluation (Table 1; excluding data completeness), identified reasons why surveillance objectives/attributes were not met and possible SWOTs. This activity included timed sessions where participants were randomly assigned by Zoom to small breakout rooms, with up to 10 participants each. In these groups, participants shared their views verbally and recorded them anonymously on an online collaborative whiteboard platform (Miro Board; https://miro.com), aiming at reducing the influence of dominant opinions. For consistency, two authors reviewed all workshop quotes, summarised them into broader thematic categories, and resolved any disagreements through discussion. Typographical errors were corrected and unclear responses were excluded. These themes then formed the multiple-choice options for the online questionnaire.

We used the Epiconcept ‘Web questionnaires for epidemiologists’ (WEPI) platform (https://www.wepi.org) to create the questionnaire. Country representatives could decide whether to submit a single response or multiple responses per country (Supplementary Table S1). The questionnaire consisted of 13 questions, each asking participants to indicate whether specific surveillance objectives and attributes were met (yes/no/unknown). If a participant indicated that an objective or attribute was not achieved, they were prompted to choose up to three main reasons from a list of six to 10 options per question. Additionally, participants were asked to identify up to three primary SWOTs from a selection of seven to 12 options. The questionnaire is provided as Supplementary Text S1. We administered the questionnaires between July and October 2023.

### Questionnaire data analysis

We extracted and consolidated the questionnaire responses to determine a single response per country, ensuring consistency and facilitating comparability across countries. For yes/no/unknown questions, the final response was determined by majority rule and considered unknown if all responses were unknown or if there was an equal split between yes and no responses, reflecting a lack of consensus. Reasons for unmet objectives and attributes were included only if the final response for the country was No. We summarised the data by counting occurrences of objectives and attributes met, reasons for unmet objectives and attributes and main SWOTs.

Additionally, we conducted two sensitivity analyses: (i) using a single-response-per-country approach but excluding those without consensus, and (ii) using a multiple-response-per-country approach.

All data analyses were performed using R statistical software (v4.4.0, R Core Team).

## Results

Since the onset of the COVID-19 pandemic, the number of countries performing SARI surveillance with support from ECDC-funded projects increased from 10 in 2020 to 27 by 2023, with seven systems under development and 20 implemented at the time of the evaluation. These implemented systems showed heterogeneity in their characteristics and a detailed description is presented in Supplementary Table S2.

### Surveillance data analysis

By October 2023, 13 of 27 countries reported data to ECDC at the European level (seven in aggregated format: Albania, Bosnia and Herzegovina, Croatia, Germany, Kosovo^§^, Montenegro and Serbia; five in case-based format: Belgium, Ireland, Malta, Romania and Spain; one in both formats: North Macedonia). The remaining 14 countries did not submit data to ECDC during the evaluation period: half were still developing their systems, while the other half had implemented systems but were working towards submission at European level.

#### Data completeness

The INFLSARIAGGR extract included 340 records from eight countries reporting aggregated data, corresponding to an overall completeness of weekly reporting of 82% (340/416 weeks). The number of weeks with data reported to ECDC varied between 33 and 52 weeks, i.e. depending on whether surveillance is active during influenza season only (week 40 to 20) or throughout the whole epidemiological year. The internal completeness was above 80% for all variables assessed, however, the number of countries reporting varied by variable, e.g. only five of eight countries reported SARI deaths (Table 2).

**Table 2:** Proportion of weekly reporting by country and internal completeness by variable for aggregated data (INFLSARIAGGR TESSy record type), 8 European countries, 2022/23

Indicator	Variable	Numerator^a^	Denominator^b^	Proportion (%)	Number of reporting countries
Completeness of weekly reporting (INFLSARIAGGR)	Country A	52	52	100	1
	Country B	33	52	63	1
	Country C	52	52	100	1
	Country D	52	52	100	1
	Country E	43	52	83	1
	Country F	42	52	81	1
	Country G	33	52	63	1
	Country H	33	52	63	1
	Overall	340	416	82	8
Internal completeness (all ages^c^) (INFLSARIAGGR)	Number of SARI hospitalisations	340	340	100	8
	Number of ICU admissions	263	274	96	6
	Number of SARI deaths	275	288	95	5
	Number of all-cause admissions	270	307	88	7
	Catchment population	307	307	100	7
	Number positive for SARS-CoV-2	298	307	97	7
	Number tested for SARS-CoV-2	297	307	97	7
	Number positive for influenza	323	340	95	8
	Number tested for influenza	331	340	97	8
	Number positive for RSV	236	255	93	6
	Number tested for RSV	249	307	81	7

ICU: intensive care unit; INFLSARIAGGR: TESSy record type to report aggregated data for SARI surveillance; RSV: respiratory syncytial virus; SARI: severe acute respiratory infection; SARS-CoV-2: severe acute respiratory syndrome coronavirus 2; TESSy: the European Surveillance System.

^a^ The numerator for the completeness of weekly reporting indicator is the number of weeks with data reported, by country and overall. The numerator for the internal data completeness indicator is the number of weeks with data reported overall, for the countries reporting the variable at least once in the year.

^b^ The denominator for the completeness of weekly reporting indicator is the number of weeks expected in the data extract, reported by country and overall. The denominator for the internal data completeness indicator is the number of weeks with data reported overall, for the countries reporting the variable at least once during the year.

^c^ All ages refer to the sum of the main age groups reported (0–4, 5–14, 15–29, 30–64 and ≥ 65 or 65–79 and ≥ 80 years).

The overall completeness of weekly reporting for both SARISURV and SARISURVDENOM record types was 86% (267/312). The number of weeks with data reported to ECDC varied between 27 and 52 weeks. Among the SARISURVDENOM records, the internal completeness was 100% for catchment population and all-cause admissions. The SARISURV extract included a total of 15,289 records from six countries. The internal completeness was above 80% for the majority of assessed variables except certain pre-existing conditions (52–75%), SARS-CoV-2 whole genome sequencing identifier (30%), SARS-CoV-2 variant (50%), influenza vaccination (54%), COVID-19 vaccination (63%) and influenza antiviral treatment (67%). The number of countries reporting varied by variable, e.g. fewer countries reported on antiviral treatment, vaccination status and sequencing results (Table 3).

**Table 3:** Proportion of weekly reporting by country and internal completeness by variable (SARISURV and SARISURVDENOM TESSy record types), 6 European countries, 2022/23

Indicator	Variable	Numerator^a^	Denominator^b^	Proportion (%)	Number of reporting countries
Completeness of weekly reporting (SARISURV and SARISURVDENOM)^c^	Country A	51	52	98	1
	Country B	52	52	100	1
	Country C	52	52	100	1
	Country D	33	52	63	1
	Country E	52	52	100	1
	Country F	27	52	52	1
	Overall	267	312	86	6
Internal completeness (SARISURVDENOM)	Catchment population (all ages)	267	267	100	6
	All-cause admissions (all ages)	164	164	100	4
Internal completeness (SARISURV)	Sex	15,257	15,289	100	6
	Age	15,142	15,289	99	6
	Date of admission to hospital	15,289	15,289	100	6
	Date of COVID-19 vaccination (fourth dose)^c^	1,327	1,327	100	4
	Date of outcome^d^	13,018	14,353	91	6
	Date of onset of symptoms	15,146	15,289	99	6
	Date of specimen collection	14,599	15,289	95	6
	Date of admission to ICU^e^	729	757	96	4
	Date of influenza vaccination^f^	675	684	99	4
	Outcome (discharged/died)	14,353	15,289	94	6
	Admission to ICU	13,813	15,289	90	6
	Respiratory support	5,144	5,199	99	4
	Fever	15,064	15,289	99	6
	Cough	14,937	15,289	98	6
	Diabetes	13,799	15,289	90	6
	Lung disease	13,707	15,289	90	6
	Pregnancy^g^	6,221	6,968	89	6
	Cardiac disease	13,390	15,289	88	6
	Kidney disease	13,317	15,289	87	6
	Hypertension	13,160	13,979	94	5
	Liver disease	12,509	14,522	86	5
	Any immunodeficiency	12,118	12,703	95	4
	Obesity	10,334	15,289	68	6
	Smoking	9,603	13,397	72	4
	Asthma	3,898	5,199	75	5
	Cancer	2,680	3,889	69	4
	Dementia	1,310	2,540	52	2
	Results for influenza	15,150	15,289	99	6
	Results for SARS-CoV-2	14,295	15,289	93	6
	Results for RSV	13,864	15,289	91	6
	SARS-CoV-2 WGS sequence identifier^h^	436	1,474	30	2
	SARS-CoV-2 variant^h^	167	337	50	3
	Influenza vaccination	2,784	5,199	54	5
	COVID-19 vaccination (fourth dose)	2,454	3,889	63	4
	Influenza antiviral treatment	1,267	1,885	67	3
	COVID-19 antiviral treatment	533	536	99	1

COVID-19: coronavirus disease; ICU: intensive care unit; RSV: respiratory syncytial virus; SARI: severe acute respiratory infection; SARISURV: TESSy record type to report case-based data for SARI surveillance; SARISURVDENOM: TESSy record type to report denominator aggregated data complementing the SARI surveillance case-based data; SARS-CoV-2: Severe acute respiratory syndrome coronavirus 2; TESSy: the European Surveillance System; WGS: whole genome sequencing.

^a^ The numerator for the completeness of weekly reporting indicator is the number of weeks with data reported by country and overall. The numerator for the internal data completeness indicator is the number of complete records reported overall, for the countries reporting the variable at least once during the year.

^b^ The denominator for the completeness of weekly reporting indicator is the number of weeks expected in the data extract, reported by country and overall. The denominator for the internal data completeness indicator is the number of weeks with data reported (SARISURVDENOM) or the number of eligible records (SARISURV) overall, for the countries reporting the variable at least once during the year.

^c^ The completeness of weekly reporting for SARISURV and SARISURVDENOM is jointly presented in this table because the two record types completement each other. Zero reporting would be a weekly record in SARISURVDENOM but no case-based reporting for that week. Incomplete reporting would be the lack of reporting in SARISURVDENOM or SARISURV for that week.

^c^ The denominator is the number of eligible records with COVID-19 vaccination (fourth dose) =  ‘yes’.

^d^ The denominator is the number of eligible records without outcome missing.

^e^ The denominator is the number of eligible records with ICU admission = ‘yes’.

^f^ The denominator is the number of eligible records with influenza vaccination = ‘yes’.

^g^ The denominator is the number of eligible records with sex = ‘female’.

^h^ The denominator is the number of eligible records with results for SARS-CoV-2 = ‘positive’.

Only six countries reported data to the ECDC during the whole year (three using aggregated and three case-based data). Further details on estimates by country are available in Supplementary Table S3 and S4.

### Online workshop and questionnaire

The target audience for the evaluation included 179 individuals from the 27 European countries implementing SARI surveillance. An overview of the workshop attendance, questionnaire responses and target audience size by country is provided in Supplementary Table S1. The attendance rate for the online workshop was 44% (79/179) with representatives from 25 countries. As a result of the online workshop, 772 quotes were categorised into 112 themes across different objectives/attributes/SWOTs. Specifically, 187 quotes related to unmet objectives were divided into 43 themes, with eight to 10 themes per objective. Another 169 quotes regarding unmet attributes were organised into 30 themes, ranging from six to nine themes per attribute. For the main SWOTs, 416 quotes were grouped into 39 themes, ranging between seven and 12 themes by SWOT. The themes identified formed the multiple-choice options in the online questionnaire. The full questionnaire, including questions and response options, is provided in Supplementary Text S1.

The country-level response rate for the questionnaire was 100%, i.e. there was at least one response from each of the 27 countries. Sixteen countries submitted a single response per country, while 11 submitted multiple responses per country. An overview of the questionnaire responses by objective/attribute is provided in Table 4. The main reasons why objectives/attributes were not met are provided in Supplementary Tables S5 and S6.

**Table 4:** Overview of questions, number of responses by objective/attribute, 27 European countries (single-response-per-country approach), 2022/23

Questions	Number of responses			Total
	Yes	No	Unknown^a^	
Does the EU-level SARI surveillance system meet its Objective 1: To monitor trends in severe respiratory infections and their impact on hospitalisations and in-hospital mortality?	23	2	2	27
Does the EU-level SARI surveillance system meet its Objective 2: To ensure the early detection and response to unusual and unexpected events caused by common or emerging respiratory pathogens?	16	7	4	27
Does the EU-level SARI surveillance system meet its Objective 3: To assess the impact of public health interventions on respiratory infections and inform disease preparedness?	16	4	7	27
Does the EU-level SARI surveillance system meet its Objective 4: To identify risk factors for severe acute respiratory infection and death?	14	9	4	27
Does the EU-level SARI surveillance system meet its Objective 5: To contribute to pathogen-specific SARI vaccine effectiveness monitoring?	15	4	8	27
Is the EU-level SARI surveillance useful?	25	1	1	27
Is the EU-level SARI surveillance acceptable?	25	1	1	27
Is the EU-level SARI surveillance system timely to meet its surveillance objectives?	16	8	3	27
Is the EU-level SARI surveillance system representative to meet its surveillance objectives?	14	6	7	27

EU: European Union; SARI: severe acute respiratory infection.

^a^ Including the responses from countries where there was no consensus.

#### Meeting surveillance objectives

The majority of country representatives (23/27) agreed European SARI surveillance achieved its objective of monitoring SARI trends (Objective 1). Nevertheless, some were undecided or felt it was not achieved, primarily because of the heterogeneity of SARI surveillance system characteristics. Approximately half of the representatives believed the remaining objectives were achieved. Barriers to meeting these objectives included insufficient representativeness/timeliness for early detection, difficulties collecting/linking data, heterogeneity in public health interventions, insufficient data on risk factors and lack of case-based data.

#### Usefulness, acceptability, timeliness and representativeness

The European SARI surveillance was perceived as being useful by most country representatives. One was unsure about its utility and another one did not find the system useful, citing issues such as lack of comparability between countries, SARI surveillance outputs not being easily available/well known and insufficient representativeness as the main reasons. Similarly, one representative found the system unacceptable, citing non-recognition of its public health importance, lack of resources and insufficient automation in data collection. While most representatives viewed the system as useful and acceptable, only half considered it timely and representative. Delays were attributed to high workloads or insufficient resources during surge periods, time-consuming case-based data collection/submission and slow/inefficient manual data submission processes. Concerns about representativeness were primarily a result of insufficient geographical coverage of participating countries.

#### Strengths, weaknesses, opportunities and threats

An overview of the main SWOTs identified is provided in Table 5. The main strength of SARI surveillance, as reported by country representatives, lies in its integrated approach, enabling the monitoring of several respiratory pathogens under a syndromic SARI case definition through laboratory testing. The other strengths included the existence of standard objectives, case definitions and protocol; being part of a European network, which is highly motivating and fosters collaboration and learning; and the fact that it covers multiple objectives. The heterogeneity of national SARI surveillance systems was the main weakness identified, followed by difficulty in maintaining the system in periods of surge activity when hospitals and clinicians are busiest, and the limited capacity at country level for automation and data linkage. The main opportunities were improvements in hospital information systems to allow data linkage/sharing, financial/technical support from ECDC and SARI being notifiable at European level as part of the case definition of respiratory viral infections. The main threats were the lack of sustainable funding, the lack of a legal framework for linking and reporting data, and changes in policy priorities that could disrupt SARI surveillance efforts.

**Table 5:** Overview of SWOTs (strengths, weaknesses, opportunities and threats) identified by country representatives, 27 European countries (single-response-per-country approach), 2022/23

SWOTs	Description	Number of responses
Strengths	Integrated surveillance with laboratory component for several respiratory pathogens	20
	Standard protocol, objectives and case definitions	19
	Covers multiple objectives (monitoring of hospitalisation trends, severity, burden of disease, early warning and preparedness, impact of public health interventions, e.g. vaccine effectiveness and vaccination programme impact)	16
	Being part of an experienced European network (collaboration, learning from each other, highly motivating)	16
	Representativeness of the system (e.g. multi-country system, large sample size)	15
	Systematic way of identifying severe patients for being sampled and monitored	11
	Timeliness of the system	10
Weaknesses	Heterogeneity of SARI surveillance system between countries (e.g. different patient recruitment protocols, different case definitions, different testing strategies)	20
	Difficult to maintain the system in periods of surge activity when hospitals and clinicians are very busy	15
	Limited capacity at country level for automation and data linkage (e.g. unavailability or difficulties linking vaccination data)	15
	Resource intensive for hospitals and national public health institutes	13
	Lack of sustainable funding to collect data, do analysis and produce outputs	12
	There is lack of interest in surveillance at the hospital level and is difficult to keep them motivated	12
	Complex system with many stakeholders making it difficult to implement and sustain	10
	Data protection issues at national level (e.g. to collect case-based data and do data linkages)	9
	Data protection issues to share data internationally	9
	SARI surveillance reporting is not mandatory	9
	SARI surveillance is not a public health priority at national level	7
Opportunities	Improvements in IT infrastructures in hospitals/laboratories to allow data linkage and data sharing	24
	Financial and technical support from ECDC-funded projects	20
	Inclusion of SARI as a notifiable disease at EU level in 2024	17
	Developments of new methods to collect data (e.g. artificial intelligence for text mining)	15
	Increased political and scientific support due to the COVID-19 pandemic	13
	Initiatives at EU level that can benefit SARI surveillance: United4Surveillance, Direct Grants, European Health Data Space	10
	New RSV vaccines may increase public and authorities’ interest in SARI surveillance	10
	Transition from universal surveillance to sentinel integrated surveillance (e.g. fear of loss of data from policymakers)	7
	New EU regulation on serious cross-border threats (2022/2371)	4
Threats	Lack of sustainable funding	20
	Lack of legal framework for reporting and linking SARI surveillance data at national level	17
	Changes in policy priorities could disrupt SARI surveillance and lead to resource re-allocation	16
	Hospitals under pressure during surge periods	15
	Laboratory data not recorded in national databases (e.g. use of point of care tests in hospitals or patient self-tests in the community)	11
	Systems implemented during the pandemic may cease to exist when COVID-19 is not considered a public health emergency of international concern (PHEIC)	10
	Overlap of projects on an EU level may lead to confusion	9
	War, humanitarian emergencies/disasters	7
	End of mandatory testing for SARS-CoV-2	7
	Duplication of activities across surveillance systems (i.e. COVID-19, Influenza, SARI)	7
	Loss of data due to overloading of the hospitals (i.e. during pandemic) or in case of failure of IT systems	6
	Lack of transparency and clarity on the use of SARI surveillance at EU level by third parties	5

COVID-19: coronavirus disease; ECDC: European Centre for Disease Prevention and Control; EU: European Union; IT: information technology; RSV: respiratory syncytial virus; SARI: severe acute respiratory infection.

### Sensitivity analysis

The results using a single-response-per-country approach while excluding those without consensus (Supplementary Table S7), and those considering multiple responses per country (Supplementary Table S8), did not differ substantially from the main analysis.

## Discussion

We found that the European-level SARI surveillance was perceived by participating countries as being able to meet its main objective of monitoring SARI trends and was considered useful and acceptable. However, only 13 of 27 countries reported data at European level and, although the overall data submitted to TESSy showed good completeness, only six countries reported throughout the year and only six reported case-based data. Internal data completeness also varied across variables and countries. Opinions on the system’s ability to meet other objectives were mixed, highlighting the need to improve timeliness, representativeness, internal data completeness, case-based data reporting, and to address data linkage challenges.

The heterogeneity of SARI surveillance systems between countries, reflected in the described variety of system characteristics, was identified as the main weakness of European SARI surveillance. This was also cited by the few countries who considered that the objective of monitoring SARI trends (Objective 1) was not met. Such heterogeneity was also mentioned as a limitation in previous evaluations of the European severe influenza [6,22,26] and COVID-19 surveillance [23]. To address this challenge, countries have adopted a standardised surveillance protocol, and unified guidance has been provided by ECDC-funded projects. Additionally, data submission at the European level is performed using a TESSy reporting protocol.

Difficulties in collecting/linking data at national level and insufficient timeliness were cited as the main reasons why SARI surveillance may fail to identify unusual presentations or emerging pathogens (Objective 2). Nonetheless, there are instances where SARI surveillance systems have successfully detected unusual events within common patterns, such as an RSV wave in Europe in the autumn of 2022 [27].

Insufficient data completeness, lack of case-based data and difficulties in linking data were perceived as the main obstacles to assessing the impact of public health interventions, identifying risk factors for SARI and monitoring pathogen-specific SARI vaccine effectiveness (Objectives 3–5). Achieving these objectives requires resource-intensive studies to collect detailed case-based information. However, such studies may not be a priority surveillance objective for countries newly implementing SARI surveillance. At the European level, only six countries report case-based data, and there was low internal completeness for key variables, such as certain pre-existing conditions, vaccination status and antiviral treatment. Data may be available at national level but are not shared at European level because of data protection concerns and data sharing policies. Nonetheless, there was high internal completeness for some pre-existing conditions and outcome variables, hence risk factor studies may be possible when data are reported. There is evidence that national SARI surveillance has been successfully used as a platform for vaccine effectiveness studies in Europe, as part of the Vaccine Effectiveness, Burden and Impact Studies (VEBIS) project [28].

Struggles with data collection/linkage at the national level and lack of case-based data were identified as barriers to achieving several surveillance objectives. Challenges included the absence of unique identifiers, data protection concerns, low clinician motivation and the lack of a legal framework. The latter two issues were also noted as a weakness and threat to the European SARI surveillance. These challenges likely vary by country and further work, such as follow-up with countries, is needed to fully understand them.

Difficulty in maintaining SARI surveillance in periods of surge activity was cited as a weakness and a reason for reduced timeliness. The timeliness of a system is associated with the system's characteristics and its acceptability [24]. Although the acceptability of the European SARI surveillance system among questionnaire respondents was high, the lack of interest at hospital level was identified as a weakness and might affect the timeliness of the system at country level. This may occur especially in systems where data collection is manual and thus more resource-intensive. Systems with a higher degree of automation are generally expected to be more timely, as they reduce the burden on hospital staff [5,16].

The insufficient geographical coverage of participating countries in Europe was pointed out as the main reason for reduced representativeness. Although many countries (n = 27) were implementing/strengthening SARI surveillance at the time of the evaluation, only a few (n = 13) report data to the ECDC. We need to understand the barriers to reporting data at European level in order to expand geographical coverage, both in terms of hospitals and countries participating in SARI surveillance. Investment in this type of surveillance also requires long-term commitment from countries.

Notable opportunities for enhancing SARI surveillance include improvements in hospital information systems, financial and technical support from ECDC, and the designation of SARI as a notifiable disease at the EU level. Initiatives such as the ECDC-funded SUREHD project are already underway to improve digitalisation and automation. Leveraging these opportunities can enhance data completeness, improve timeliness and expand the geographical coverage of SARI surveillance systems.

We have identified several limitations in our evaluation of the European SARI surveillance system. Firstly, the assessment of objectives and attributes at the European level (except for data completeness) primarily relied on subjective perceptions and opinions. Capturing stakeholder views is vital at this stage of implementation, as the success of the European SARI surveillance depends on collaborative efforts between countries and ECDC. Both single- and multiple-response questionnaire approaches had limitations. The single-response approach ensured consistency and facilitated comparability across countries, but may have missed divergent views within a country. In contrast, the multiple-response approach captured a broader range of perspectives but risked diluting national consensus and giving disproportionate weight to countries with more responses. Sensitivity analyses revealed no significant differences between the approaches. Additionally, as the questionnaires were anonymous, we could not follow up with respondents to resolve any lack of consensus within a country. Secondly, the overall data completeness assessment represents an aggregated measure for all countries and the entire year at a single point in time, which may not equally represent all countries and or capture seasonal variations in data completeness. Thirdly, this evaluation was conducted during a post-pandemic period with many surveillance systems still under redevelopment and before the implementation of changes to TESSy reporting protocols and launch of the ERVISS dashboard. Consequently, our findings may not fully reflect the system’s performance in a non-pandemic context. A notable proportion of countries (up to 30%) expressed uncertainty when queried about the system’s objectives and attributes at the European level. This uncertainty likely stems from the fact that this was the first evaluation of its kind at the European level, and that detailed information about surveillance objectives and attributes is limited in publicly available routine outputs. Because of the anonymous nature of the questionnaire, we were unable to follow up with respondents to clarify the reasons for this uncertainty. Fourthly, this evaluation excluded nine EU/EEA countries that were not part of ECDC-funded projects to strengthen SARI surveillance. Finally, the evaluation team included members who also served as national representatives for three of the 27 countries. While this overlap provided valuable expertise, it may have introduced bias. To mitigate this, an anonymous questionnaire was used, with responses consolidated into a single response per country to minimise individual influence.

Our evaluation has generated several priority recommendations to improve the European SARI surveillance system in the short term. Improving representation and the number of countries and sites participating is a longer-term recommendation (Box). Recent country-specific SARI evaluations identified similar areas for improvement and concurred with many of these recommendations [29,30].

Box **Overview of areas for improvement and recommendations for European SARI surveillance**

**Surveillance system heterogeneity**

- Promote harmonisation of SARI surveillance systems by sharing experiences and standardised protocols
- Investigate how surveillance system heterogeneity affects data comparability at European level
- Account for system heterogeneity when reporting/interpreting surveillance indicators by stratification or weighting

**Data completeness**

- Support countries in transitioning from seasonal to full-year reporting
- Identify the reasons for the lack of reporting or low completeness of certain variables and clarify their importance/rationale
- Provide regular feedback on surveillance objectives and attributes to improve system performance
- Identify and address the challenges to collecting and reporting case-based data
- Investigate the potential for selection bias when specific information is available only from certain cases or countries

**Timeliness**

- Conduct a quantitative assessment of timeliness at the country level to determine whether specific surveillance system characteristics contribute to its variation

**Representativeness**

- Explore and address barriers to reporting data at European level
- Strengthen country-level support to expand the number of reporting sites within each country and increase the number of countries submitting SARI surveillance data at European level

**Future evaluations**

- Use country-specific weekly data extractions rather than a single end-of-season extraction to better assess seasonal patterns, country-level differences and temporal trends in data completeness
- Incorporate quantitative assessments at the country level using detailed data and external reference databases to evaluate key attributes (e.g. assess concordance between primary care acute respiratory illness incidence and hospital-based SARI incidence to evaluate the objective of monitoring SARI trends)
- Enhance qualitative assessments by incorporating follow-up mechanisms, such as structured interviews or additional survey questions, to clarify uncertain or negative responses
- Aim to include all EU/EEA countries to enable a more comprehensive assessment

EU/EEA: European Union/European Economic Area; SARI: severe acute respiratory infection.

## Conclusions

Many countries in Europe implemented or enhanced their SARI surveillance systems between 2020 and 2023. However, only a few reported data at the European level during the 2022/23 epidemiological year. While the SARI surveillance system at European level was considered useful, acceptable and effective at monitoring SARI trends, improvements are still needed. To enable the European SARI surveillance system to meet all its objectives and to improve both timeliness and representativeness, further country-level investment in SARI surveillance is essential. We recommend focusing on strategies to enhance data completeness, improve digitalisation/automation and expand geographical coverage. Future evaluations should incorporate quantitative assessments of key attributes at country level.

## Data Availability

Data are available upon reasonable request to the corresponding author.

## § Note

This designation is without prejudice to positions on status, and is in line with United Nations Security Council Resolution 1244/99 and the International Court of Justice Opinion on the Kosovo Declaration of Independence.

## Supplementary Data

446000 Supplement
