# Supplementary material for: Evaluation of European severe acute respiratory infection (SARI) surveillance, 27 European countries, 2022/23
Source: Euro Surveill. 2025 May 22;30(20):2400655. doi: 10.2807/1560-7917.ES.2025.30.20.2400655 (PMC12105088; doi:10.2807/1560-7917.ES.2025.30.20.2400655)
Supplement: Supplement [file 24-00655_MARQUES_Supplement.pdf]

Supplementary material

This supplementary material is hosted by *Eurosurveillance* as supporting information alongside the article **"Evaluation of European severe acute respiratory infection (SARI) surveillance, 27 European countries, 2022/23"**, on behalf of the authors, who remain responsible for the accuracy and appropriateness of the content. The same standards for ethics, copyright, attributions and permissions as for the article apply. Supplements are not edited by *Eurosurveillance* and the journal is not responsible for the maintenance of any links or email addresses provided therein.

Contents

Supplementary Table S1 - Overview of the target audience, workshop attendance and questionnaire responses by country, 27 European countries, 2022/23... 2

Supplementary Table S2 – Description of the characteristics of SARI surveillance systems strengthened through ECDC-funded projects, 27 European countries, 2022/23........ 3

Supplementary Table S3 - Internal completeness (all ages) by variable and country for aggregated data (INFLSARIAGGR), 8 European countries, 2022/23...... 6

Supplementary Table S4 - Internal completeness by variable and country for SARISURV and SARISURVDENOM TESSy record types, 6 European countries,..... 7

Supplementary Table S5 - Overview of main reasons why objectives were not met, number of responses by objective, 27 European countries, 2022/23......... 9

Supplementary Table S6 - Overview of main reasons why attributes were not achieved, number of responses by attribute, 27 European countries, 2022/23.. 11

Supplementary Table S7 - Overview of questions, number of responses by objective/attribute, 27 European countries, 2022/23 (single-response-per-country approach while excluding those without consensus)........ 12

Supplementary Table S8 - Overview of questions, number of responses by objective/attribute, 27 European countries, 2022/23 (multiple-response-per-country approach)........ 13

Supplementary Text S1 - Online questionnaire to assess surveillance attributes and identify SWOTs, 27 European countries, 2022/23........14

Supplementary Table S1 - Overview of the target audience, workshop attendance and questionnaire responses by country, 27 European countries, 2022/23

Country	Evaluation target audience	Workshop attendance	Questionnaire responses
Albania	6	4	2
Austria	6	3	2
Belgium	7	4	1
Bosnia & Herzegovina	13	2	1
Bulgaria	6	3	3
Croatia	14	8	1
Cyprus	4	1	1
Czechia	3	2	1
Denmark	5	2	1
Estonia	3	2	1
France	11	3	2
Germany	6	2	1
Iceland	4	2	1
Ireland	7	6	6
Italy	4	0	1
Kosovo [§]	6	2	1
Lithuania	8	2	1
Luxembourg	4	2	1
Malta	5	2	4
Montenegro	5	1	2
Netherlands	6	5	1
North Macedonia	10	1	3
Norway	4	2	2
Portugal	13	9	6
Romania	2	0	2
Serbia	7	3	1
Spain	10	6	1
Total	179	79	50

SARI: severe acute respiratory infection.

[§] This designation is without prejudice to positions on status, and is in line with United Nations Security Council Resolution 1244/99 and the International Court of Justice Opinion on the Kosovo Declaration of Independence.

Supplementary Table S2 – Description of the characteristics of SARI surveillance systems strengthened through ECDC-funded projects, 27 European countries, 2022/23

Note: These system characteristics were obtained through a desk review of country-specific protocols and annual reports from ECDC-funded SARI surveillance projects. It is important to note that the information reflects the "date of information update" indicated in the tables (e.g., February, March, or May 2024) and relates to the components of SARI surveillance that were implemented or strengthened as part of these ECDC-funded projects. This description is not exhaustive, as countries may have other complementary components of SARI surveillance not covered here. As countries continue to implement and strengthen their SARI surveillance systems, these characteristics may change over time. In some cases, countries are implementing two systems (e.g., one sentinel and one comprehensive, or one with active data collection and another with passive data collection). These are denoted as (a) and (b) in the tables for Bulgaria, France and Portugal.

Country	Albania (AL)	Austria (AT)	Belgium (BE)	Bosnia & Herzegovina (BH)	Bulgaria (BU) (a)	Bulgaria (BU) (b)
**Implementation status**	Implemented	Implemented	Implemented	Implemented	Implemented	Under development
**Implementation year**	2008	2023	2012	2016	2017	NA
**Reporting level**	European	European	European	European	National	NA
**TESSy reporting start [a]**	2020-W40	2023-W40	2021-W04	2020-W40	NA	NA
**TESSy reporting format**	Aggregated	Aggregated	Case-based	Aggregated	NA	NA
**System design [b]**	Sentinel	Comprehensive	Sentinel	Sentinel	Sentinel	Comprehensive
**Number of hospitals**	14	154	10	3	4	2
**Catchment population [c]**	2,761,785	NA	1,857,579	1,000,000	504,765	572,276
**Country population**	2,761,785	NA	11,697,557	3,185,073	6,447,710	6,447,710
**System coverage (%) [d]**	100	95%	16	31	8	9
**Type of data collection [e]**	Active	Passive	Active	Active	Active	Passive
**Case identification**	Symptoms	Diagnostic codes	Symptoms	Symptoms	Symptoms	Diagnostic codes
**Pathogen testing**	Systematic	At clinician discretion	Systematic	Systematic	At clinician discretion	At clinician discretion
**Temporal continuity [f]**	All-year	All-year	All-year	Seasonal	All-year	All-year
**Format of data collection**	Case-based	Aggregated	Case-based	Aggregated	Case-based	Case-based
**Date of information update**	March 2024	February 2024	May 2024	March 2024	May 2024	May 2024

NA: Not applicable; **SARI:** Severe acute respiratory infection; **TESSy**: the European Surveillance System.

[a] TESSy reporting start: first year-week of reporting to TESSy after start of ECDC SARI surveillance projects (2020-W40).
[b] System design: comprehensive if all hospitals from the region or country are included in the surveillance system; sentinel if a sample of hospitals from a region or country is included in the surveillance system.
[c] Catchment population: population covered by the surveillance system.
[d] System coverage: the catchment population is the numerator and the country population the denominator.
[e] Type of data collection: active if the main purpose of data collection is for SARI surveillance, e.g. using questionnaires to collect all information required on SARI cases. Passive if data are collected primarily for other purposes but can be used for SARI surveillance, e.g. using linkage of data from hospital, laboratory, population and mortality databases to gather the required information on SARI cases.
[f] Temporal continuity: period of activity of the surveillance system, i.e. only seasonal or all-year-round.

Country	Croatia (HR)	Cyprus (CY)	Czechia (CZ)	Denmark (DK)	Estonia (EE)	France (FR) (a)	France (FR) (b)
**Implementation status**	Implemented	Implemented	Under development	Implemented	Under development	Implemented	Under development
**Implementation year**	2021	2023	NA	2023	NA	2021	NA
**Reporting level**	European	European	NA	National	NA	National	NA
**TESSy reporting start [a]**	2021-W05	2024-W01	NA	NA	NA	NA	NA
**TESSy reporting format**	Aggregated	Aggregated	NA	NA	NA	NA	NA
**System design [b]**	Sentinel	Sentinel	Comprehensive	Comprehensive	Comprehensive	Sentinel	Sentinel
**Number of hospitals**	1	6	NA	NA	NA	8	15 - 20
**Catchment population [c]**	447,747	64,274	10,900,555	5,995,628	NA	To be estimated	To be estimated
**Country population**	3,888,529	918,100	10,900,555	5,995,628	NA	NA	NA
**System coverage (%) [d]**	12	26	100	100	NA	To be estimated	To be estimated
**Type of data collection [e]**	Active	Passive	Passive	Passive	Passive	Active	Passive
**Case identification**	Symptoms	Other codes	Diagnostic codes	Diagnostic codes	Diagnostic codes	Symptoms	To be defined
**Pathogen testing**	Systematic	At clinician discretion	At clinician discretion	At clinician discretion	At clinician discretion	Systematic	To be defined
**Temporal continuity [f]**	All-year	All-year	All-year	All-year	All-year	Seasonal	All-year
**Format of data collection**	Case-based	Case-based	Case-based	Case-based	Both	Case-based	Aggregated
**Date of information update**	May 2024	February 2024	February 2024	May 2024	February 2024	May 2024	February 2024

Country	Germany (DE)	Iceland (IS)	Ireland (IE)	Italy (IT)	Kosovo (XK) [§]	Lithuania (LT)	Luxembourg (LU)
**Implementation status**	Implemented	Under development	Implemented	Under development	Implemented	Under development	Under development
**Implementation year**	2015	NA	2021	NA	2014	NA	NA
**Reporting level**	European	NA	European	NA	European	NA	NA
**TESSy reporting start [a]**	2020-W40	NA	2021-W27	NA	2022-W40	NA	NA
**TESSy reporting format**	Aggregated	NA	Case-based	NA	Aggregated	NA	NA
**System design [b]**	Sentinel	Comprehensive	Sentinel	Comprehensive	Sentinel	Sentinel	Comprehensive
**Number of hospitals**	70	NA	1	NA	5	7	4
**Catchment population [c]**	4,423,024	NA	316,230	NA	885,001	664,917	672 050
**Country population**	84,358,845	NA	5,149,139	NA	1,873,000	2,886,515	672 050
**System coverage (%) [d]**	5	NA	6	NA	47	23	100%
**Type of data collection [e]**	Passive	Passive	Active	Passive	Active	Active	Passive
**Case identification**	Diagnostic codes	Diagnostic codes	Symptoms	Diagnostic codes	Symptoms	Symptoms and Diagnostic codes	Diagnostic codes
**Pathogen testing**	Systematic	At clinician discretion	Systematic	At clinician discretion	Systematic	Systematic	At clinician discretion
**Temporal continuity [f]**	All-year	All-year	All-year	All-year	Seasonal	All-year	All-year
**Format of data collection**	Case-based	Case-based	Case-based	Case-based	Aggregated	Aggregated	Case-based
**Date of information update**	May 2024	February 2024	May 2024	May 2024	March 2024	May 2024	May 2024

[§] This designation is without prejudice to positions on status, and is in line with United Nations Security Council Resolution 1244/99 and the International Court of Justice Opinion on the Kosovo Declaration of Independence.

Country	Malta (MT)	Montenegro (ME)	Netherlands (NL)	North Macedonia (MK)	Norway (NO)
**Implementation status**	Implemented	Implemented	Under development	Implemented	Implemented
**Implementation year**	2020	2014	NA	2014	2021
**Reporting level**	European	European	NA	European	National
**TESSy reporting start [a]**	2021-W05	2021-W40	NA	2021-W40	NA
**TESSy reporting format**	Case-based	Aggregated	NA	Both	NA
**System design [b]**	Comprehensive	Sentinel	Comprehensive	Sentinel	Comprehensive
**Number of hospitals**	1	3	NA	6	NA
**Catchment population [c]**	563 443	620,029	NA	1,050,428	5,550,203
**Country population**	563 443	620,029	NA	1,836,713	5,550,203
**System coverage (%) [d]**	100%	100	NA	57	100
**Type of data collection [e]**	Active	Active	Passive	Active	Passive
**Case identification**	Symptoms	Symptoms	Other codes	Symptoms	Diagnostic codes
**Pathogen testing**	At clinician discretion	Systematic	At clinician discretion	Systematic	At clinician discretion
**Temporal continuity [f]**	All-year	Seasonal	All-year	All-year	All-year
**Format of data collection**	Case-based	Aggregated	Both	Both	Case-based
**Date of information update**	May 2024	March 2024	February 2024	March 2024	May 2024

Country	Portugal (PT) (a)	Portugal (PT) (b)	Romania (RO)	Serbia (RS) [g]	Spain (ES)
**Implementation status**	Implemented	Under development	Implemented	Implemented	Implemented
**Implementation year**	2020	NA	2009	2009	2020
**Reporting level**	National	NA	European	European	European
**TESSy reporting start [a]**	NA	NA	2021-W46	2020-W40 [a]	2021-W40
**TESSy reporting format**	NA	NA	Case-based	Aggregated	Syndromic surveillance: Aggregated<br>Systematic surveillance: Case-based
**System design [b]**	Sentinel	Comprehensive	Sentinel	Sentinel	Sentinel
**Number of hospitals**	3	NA	19	9	Syndromic surveillance: 60<br>Systematic surveillance: 38
**Catchment population [c]**	777,524	NA	2,606,589	~ 4.6 million	Syndromic surveillance: ~ 15.6 million<br>Systematic surveillance: ~ 12 million
**Country population**	10,447,220	NA	19,599,935	6,664,449	47,432,893
**System coverage (%) [d]**	7	NA	13	70	Syndromic surveillance: 33<br>Systematic surveillance: 25
**Type of data collection [e]**	Passive	Passive	Active	Active	Active
**Case identification**	Diagnostic codes	Diagnostic codes	Symptoms	Symptoms	Diagnostic codes + Diagnostic impressions + Manual revision of clinical records
**Pathogen testing**	At clinician discretion	At clinician discretion	Systematic	At clinician discretion	Syndromic surveillance: None<br>Systematic surveillance: Systematic
**Temporal continuity [f]**	All-year	All-year	Seasonal	All-year	All-year
**Format of data collection**	Case-based	Both	Case-based	Aggregated	Syndromic surveillance: Case-based<br>Systematic surveillance: Case-based
**Date of information update**	May 2024	February 2024	May 2024	March 2024	May 2024

[g] Serbia has been reporting SARI surveillance data to TESSy since the 2014-2015 influenza season.

Supplementary Table S3 - Internal completeness (all ages) by variable and country for aggregated data (INFLSARIAGGR), 8 European countries, 2022/23

	Country A			Country B			Country C			Country D			Country E			Country F			Country G			Country H		
**Variable**	**N**	**D**	**%**	**N**	**D**	**%**	**N**	**D**	**%**	**N**	**D**	**%**	**N**	**D**	**%**	**N**	**D**	**%**	**N**	**D**	**%**	**N**	**D**	**%**
Number of SARI hospitalisations	52	52	**100**	33	33	**100**	52	52	**100**	52	52	**100**	43	43	**100**	42	42	**100**	33	33	**100**	33	33	**100**
Number of ICU admissions	50	52	**96**	NA	NA	NA	52	52	**100**	52	52	**100**	43	43	**100**	33	42	**79**	NA	NA	NA	33	33	**100**
Number of SARI deaths	NA	NA	NA	31	33	**94**	52	52	**100**	52	52	**100**	43	43	**100**	33	42	**79**	31	33	**94**	33	33	**100**
Number of all-cause admissions	15	52	**29**	33	33	**100**	52	52	**100**	52	52	**100**	43	43	**100**	42	42	**100**	33	33	**100**	NA	NA	NA
Catchment population	52	52	**100**	33	33	**100**	52	52	**100**	52	52	**100**	43	43	**100**	42	42	**100**	NA	NA	NA	33	33	**100**
Number positive for SARS-CoV-2	52	52	**100**	33	33	**100**	52	52	**100**	52	52	**100**	43	43	**100**	33	42	**79**	NA	NA	NA	33	33	**100**
Number tested for SARS-CoV-2	52	52	**100**	33	33	**100**	52	52	**100**	52	52	**100**	43	43	**100**	33	42	**79**	NA	NA	NA	32	33	**97**
Number positive for influenza	52	52	**100**	33	33	**100**	48	52	**92**	52	52	**100**	43	43	**100**	33	42	**79**	31	33	**94**	31	33	**94**
Number tested for influenza	52	52	**100**	33	33	**100**	52	52	**100**	52	52	**100**	43	43	**100**	33	42	**79**	33	33	**100**	33	33	**100**
Number positive for RSV	NA	NA	NA	33	33	**100**	42	52	**81**	52	52	**100**	43	43	**100**	33	42	**79**	NA	NA	NA	33	33	**100**
Number tested for RSV	4	52	**8**	33	33	**100**	52	52	**100**	52	52	**100**	43	43	**100**	33	42	**79**	NA	NA	NA	32	33	**97**

%: proportion; **D**: denominator; the number of weeks with data reported for countries that reported the variable at least once in the year (those not reporting at all are marked “NA”); **ICU**: intensive care unit; **NA**: Not applicable, i.e. the country did not report this variable once in the year; **N**: numerator; the number of weeks with data reported for countries that reported the variable at least once in the year (those not reporting at all are marked “NA”); **RSV**: respiratory syncytial virus; **SARI**: severe acute respiratory infection; **SARS-CoV-2**: Severe acute respiratory syndrome – coronavirus 2; **TESSy**: the European Surveillance System.

Note: **All ages**: refers to the sum of the main age groups reported (00–04, 05–14, 15–29, 30–64, and 65+ or 65–79 and 80+ years old); **INFLSARIAGGR**: TESSy record type to report aggregated data for SARI surveillance;

Supplementary Table S4 - Internal completeness by variable and country for SARISURV and SARISURVDENOM TESSy record types, 6 European countries, 2022/23

	Country A			Country B			Country C			Country D			Country E			Country F		
**Variable**	**N**	**D**	**%**	**N**	**D**	**%**	**N**	**D**	**%**	**N**	**D**	**%**	**N**	**D**	**%**	**N**	**D**	**%**
Catchment population (all ages) (SARISURVDENOM)	51	51	**100**	52	52	**100**	52	52	**100**	33	33	**100**	27	27	**100**	52	52	**100**
All-cause admissions (all ages) (SARISURVDENOM)	NA	NA	NA	52	52	**100**	52	52	**100**	33	33	**100**	27	27	**100**	NA	NA	NA
Sex	1,282	1,310	**98**	767	767	**100**	2,004	2,004	**100**	582	582	**100**	536	536	**100**	10,086	10,090	**100**
Age	1,163	1,310	**89**	767	767	**100**	2,004	2,004	**100**	582	582	**100**	536	536	**100**	10,090	10,090	**100**
Date of admission to hospital	1,310	1,310	**100**	767	767	**100**	2,004	2,004	**100**	582	582	**100**	536	536	**100**	10,090	10,090	**100**
Date of COVID-19 vaccination (fourth dose) [a]	NA	NA	NA	575	575	**100**	745	745	**100**	6	6	**100**	1	1	**100**	NA	NA	NA
Date of outcome [b]	865	884	**98**	728	728	**100**	2,002	2,004	**100**	582	582	**100**	60	536	**11**	8,771	9,619	**91**
Date of onset of symptoms	1,310	1,310	**100**	767	767	**100**	2,004	2,004	**100**	566	582	**97**	535	536	**100**	9,964	10,090	**99**
Date of specimen collection	1,310	1,310	**100**	749	767	**98**	1,672	2,004	**83**	582	582	**100**	534	536	**100**	9,752	10,090	**97**
Date of admission to ICU [c]	NA	NA	NA	40	40	**100**	63	63	**100**	NA	NA	NA	135	147	**92**	491	507	**97**
Date of influenza vaccination [d]	NA	NA	NA	8	8	**100**	619	619	**100**	14	14	**100**	34	43	**79**	NA	NA	NA
Outcome (discharged/died)	884	1,310	**67**	728	767	**95**	2,004	2,004	**100**	582	582	**100**	536	536	**100**	9,619	10,090	**95**
Admission to ICU	81	1,310	**6**	740	767	**96**	2,004	2,004	**100**	579	582	**99**	536	536	**100**	9,873	10,090	**98**
Respiratory support	1,310	1,310	**100**	724	767	**94**	1,993	2,004	**99**	582	582	**100**	535	536	**100**	NA	NA	NA
Fever	1,310	1,310	**100**	766	767	**100**	1,986	2,004	**99**	582	582	**100**	536	536	**100**	9,884	10,090	**98**
Cough	1,310	1,310	**100**	767	767	**100**	1,840	2,004	**92**	582	582	**100**	536	536	**100**	9,902	10,090	**98**
Diabetes	1,310	1,310	**100**	766	767	**100**	977	2,004	**49**	582	582	**100**	536	536	**100**	9,628	10,090	**95**
Lung disease	1,310	1,310	**100**	767	767	**100**	750	2,004	**37**	582	582	**100**	536	536	**100**	9,762	10,090	**97**
Pregnancy [e]	571	571	**100**	405	405	**100**	849	905	**94**	255	255	**100**	267	269	**99**	3,874	4,563	**85**
Cardiac disease	1,310	1,310	**100**	767	767	**100**	277	2,004	**14**	582	582	**100**	536	536	**100**	9,918	10,090	**98**
Kidney disease	1,310	1,310	**100**	767	767	**100**	663	2,004	**33**	582	582	**100**	536	536	**100**	9,459	10,090	**94**
Hypertension	NA	NA	NA	766	767	**100**	1,296	2,004	**65**	582	582	**100**	536	536	**100**	9,980	10,090	**99**
Liver disease	1,310	1,310	**100**	NA	NA	NA	584	2,004	**29**	582	582	**100**	536	536	**100**	9,497	10,090	**94**
Any immunodeficiency	1,310	1,310	**100**	766	767	**100**	NA	NA	NA	NA	NA	NA	536	536	**100**	9,506	10,090	**94**
Obesity	1,310	1,310	**100**	693	767	**90**	594	2,004	**30**	582	582	**100**	517	536	**96**	6,638	10,090	**66**
Smoking	NA	NA	NA	650	767	**85**	1,551	2,004	**77**	NA	NA	NA	532	536	**99**	6,870	10,090	**68**
Asthma	1,310	1,310	**100**	765	767	**100**	706	2,004	**35**	582	582	**100**	535	536	**100**	NA	NA	NA
Cancer	NA	NA	NA	764	767	**100**	798	2,004	**40**	582	582	**100**	536	536	**100**	NA	NA	NA
Dementia	NA	NA	NA	NA	NA	NA	774	2,004	**39**	NA	NA	NA	536	536	**100**	NA	NA	NA
Results for influenza	1,310	1,310	**100**	765	767	**100**	2,004	2,004	**100**	582	582	**100**	536	536	**100**	9,935	10,090	**98**
Results for SARS-CoV-2	1,310	1,310	**100**	767	767	**100**	2,004	2,004	**100**	579	582	**99**	536	536	**100**	9,087	10,090	**90**
Results for RSV	1,310	1,310	**100**	767	767	**100**	2,004	2,004	**100**	582	582	**100**	535	536	**100**	8,665	10,090	**86**
SARS-CoV-2 WGS sequence identifier [f]	NA	NA	NA	135	156	**87**	NA	NA	NA	NA	NA	NA	NA	NA	NA	301	1,318	**23**
SARS-CoV-2 variant [f]	NA	NA	NA	137	156	**88**	21	92	**23**	NA	NA	NA	9	89	**10**	NA	NA	NA
Influenza vaccination	1,064	1,310	**81**	8	767	**1**	619	2,004	**31**	582	582	**100**	511	536	**95**	NA	NA	NA
COVID-19 vaccination (fourth dose)	NA	NA	NA	591	767	**77**	745	2,004	**37**	582	582	**100**	536	536	**100**	NA	NA	NA
Influenza antiviral treatment	NA	NA	NA	722	767	**94**	NA	NA	NA	12	582	**2**	533	536	**99**	NA	NA	NA
COVID-19 antiviral treatment	NA	NA	NA	NA	NA	NA	NA	NA	NA	NA	NA	NA	533	536	**99**	NA	NA	NA

%: proportion; **COVID-19**: Coronavirus disease 2019; **D**: denominator; the number of weeks with data reported (SARISURVDENOM) or the number of eligible records (SARISURV), for countries that reported the variable at least once in the year (those not reporting at all are marked "NA"); **ICU**: intensive care unit; **N**: numerator; the number of complete records reported, for countries that reported the variable at least once in the year (those not reporting at all are marked "NA"); **RSV**: respiratory syncytial virus; **SARI**: severe acute respiratory infection; **SARISURV**: TESSy record type to report case-based data for SARI surveillance; **SARISURVDENOM**: TESSy record type to report denominator aggregated data complementing the SARI surveillance case-based data; **SARS-CoV-2**: Severe acute respiratory syndrome – coronavirus 2; **TESSy**: the European Surveillance System; **WGS**: whole genome sequencing.

[a] The denominator is the number of eligible records with COVID-19 vaccination (Fourth dose) = "yes"
[b] The denominator is the number of eligible records without outcome missing
[c] The denominator is the number of eligible records with ICU admission = "yes"
[d] The denominator is the number of eligible records with influenza vaccination = "yes"
[e] The denominator is the number of eligible records with sex = "female"
[f] The denominator is the number of eligible records with results for SARS-CoV-2 = "positive"

Supplementary Table S5 - Overview of main reasons why objectives were not met, number of responses by objective, 27 European countries, 2022/23

Objective	Reason why objectives were not met	Number of responses	Number of countries that responded the objective was "not met"
Objective 1	Lack of case-based data	2	2
	Laboratory data not recorded in national databases (e.g. use of point of care tests in hospitals or patient self-tests in the community)	2	2
	Heterogeneity of SARI surveillance system between countries (e.g. different patient recruitment protocols, different case definitions, different testing strategies)	2	2
	Difficulties collecting/linking data at national level (e.g. lack of unique identifier, data protection issues, low clinician motivation, lack of legal framework)	1	2
	Insufficient data completeness/validity on outcomes	1	2
	High workload or not enough resources	1	2
	Lack of automation of systems	1	2
	Insufficient capacity for virological subtyping/characterisation/sequencing	0	2
	Difficulties sharing data to the European level (e.g. data protection issues)	0	2
Objective 2	Difficulties collecting/linking data at national level (e.g. lack of unique identifier, data protection issues, low clinician motivation, lack of legal framework)	4	7
	Insufficient timeliness (e.g. to allow early detection)	4	7
	Lack of comparable historical data (e.g. to ascertain what is unusual/unexpected)	3	7
	Existing case definitions may miss unusual presentations or emerging respiratory pathogens	3	7
	Insufficient representativeness (e.g. to allow early detection)	3	7
	Lack of case-based data	1	7
	Laboratory data not recorded in national databases (e.g. use of point of care tests in hospitals or patient self-tests in the community)	1	7
	Insufficient capacity for virological subtyping/characterisation/sequencing	1	7
	Difficulties sharing data to the European level (e.g. data protection issues)	1	7
	Insufficient flexibility (e.g. to detect unusual presentations or emerging respiratory pathogens)	0	7
Objective 3	Heterogeneity of public health interventions between countries	4	4
	Difficulties collecting/linking data at national level (e.g. lack of unique identifier, data protection issues, low clinician motivation, lack of legal framework)	3	4
	Insufficient data completeness/validity on public health interventions (e.g. vaccination status, antiviral prophylaxis or treatment)	3	4
	Lack of case-based data	2	4
	Insufficient data completeness/validity on outcomes	2	4
	Laboratory data not recorded in national databases (e.g. use of point of care tests in hospitals or patient self-tests in the community)	1	4
	Insufficient capacity for virological subtyping/characterisation/sequencing	1	4
	Difficulties sharing data to the European level (e.g. data protection issues)	1	4
Objective 4	Insufficient data completeness/validity on risk factors	8	9
	Lack of case-based data	6	9

	Lack of details on variables to collect risk factors (e.g. definitions of preconditions)	5	9
	Difficulties collecting/linking data at national level (e.g. lack of unique identifier, data protection issues, low clinician motivation, lack of legal framework)	4	9
	Insufficient data completeness/validity on outcomes	3	9
	Laboratory data not recorded in national databases (e.g. use of point of care tests in hospitals or patient self-tests in the community)	1	9
	Difficulties sharing data to the European level (e.g. data protection issues)	1	9
	Insufficient capacity for virological subtyping/characterisation/sequencing	0	9
Objective 5	Lack of case-based data	4	4
	Difficulties collecting/linking data at national level (e.g. lack of unique identifier, data protection issues, low clinician motivation, lack of legal framework)	3	4
	Insufficient data completeness/validity on outcomes	3	4
	Insufficient data completeness/validity on vaccination status	3	4
	Heterogeneity of laboratory tests being used (e.g. PCR vs RADT)	1	4
	Laboratory data not recorded in national databases (e.g. use of point of care tests in hospitals or patient self-tests in the community)	0	4
	Insufficient capacity for virological subtyping/characterisation/sequencing	0	4
	Difficulties sharing data to the European level (e.g. data protection issues)	0	4

PCR: polymerase chain reaction; **RADT**: rapid antigen diagnostic test; **SARI**: severe acute respiratory infection

Supplementary Table S6 - Overview of main reasons why attributes were not achieved, number of responses by attribute, 27 European countries, 2022/23

Attribute	Reason why attributes were not achieved	Number of responses	Number of countries that responded the attribute was "not achieved"
Usefulness	Lack of comparability between hospitals/countries (different case definitions, methodologies)	1	1
	SARI surveillance outputs are not easily available/not well known	1	1
	Insufficient representativeness (e.g. results may reflect what is happening in larger countries)	1	1
	Does not meet any of the surveillance objectives	0	1
	Lack of case-based data	0	1
	SARI surveillance data are not fully used	0	1
	Insufficient acceptability (e.g. to data are not collected/shared due to lack of clinician motivation, increased workload or public health importance of SARI surveillance not being recognised)	0	1
Acceptability	The public health importance of SARI surveillance is not recognised at national level	1	1
	Lack of resources	1	1
	Lack of automation in data collection process	1	1
	The public health importance of SARI surveillance is not recognised at hospital level	0	1
	Increased workload for hospital staff / conflicting priorities	0	1
	Ethical issues to collect/share data (e.g. SARI surveillance considered as research, requirements for informed consent)	0	1
	Data protection to collect/share data (e.g. data linkage, data sharing at EU level, risk of patient identification due to small counts)	0	1
	Weekly data submission is challenging	0	1
Timeliness	Case-based data collection and submission is time consuming	5	8
	Manual data extraction and submission is slow/inefficient	5	8
	High workload or insufficient resources to collect/submit data (especially during surge activity periods)	5	8
	Delays in laboratory testing	3	8
	Requirement to wait for discharge to submit data (e.g. if submission depends on discharge ICD-10 codes or discharge outcomes)	3	8
	High number of variables to be collected	2	8
Representativeness	Insufficient geographical representativeness (e.g. poor coverage of participating countries)	6	6
	Insufficient age group representativeness (e.g. focus is on adult age groups)	3	6
	Data collection and sharing is limited by ethical and data protection issues	3	6
	Insufficient urban/rural representativeness (e.g. focus is on urban areas)	2	6
	Lack of harmonised SARI patient recruitment protocols	2	6
	Lack of harmonised SARI case definitions	2	6
	Insufficient socio-economic group or private/public hospital representativeness	1	6
	Insufficient representativeness of severity (e.g. the system focuses on more severe hospitalisations, i.e. ICU admissions)	1	6
	Insufficient representativeness of mortality (e.g. the system misses those who died within 24h of admission or after discharge)	1	6

EU: European Union; **ICD**: international classification of diseases; **ICU**: Intensive care unit; **SARI**: severe acute respiratory infection

Supplementary Table S7 - Overview of questions, number of responses by objective/attribute, 27 European countries, 2022/23 (single-response-per-country approach while excluding those without consensus)

Questions	Yes	No	Unknown [a]	Total	No consensus [b]
Does the EU-level SARI surveillance system meet its **objective 1**: To monitor trends in severe respiratory infections and their impact on hospitalisations and in-hospital mortality?	23	2	1	26	1
Does the EU-level SARI surveillance system meet its **objective 2**: To ensure the early detection and response to unusual and unexpected events caused by common or emerging respiratory pathogens?	16	7	3	26	1
Does the EU-level SARI surveillance system meet its **objective 3:** To assess the IMPACT of public health interventions on respiratory infections and inform disease preparedness?	16	4	6	26	1
Does the EU-level SARI surveillance system meet its **objective 4**: To identify risk factors for severe acute respiratory infection and death?	14	9	2	25	2
Does the EU-level SARI surveillance system meet its **objective 5**: To contribute to pathogen-specific SARI vaccine effectiveness monitoring?	15	4	7	26	1
Is the EU-level SARI surveillance **useful**?	25	1	1	27	0
Is the EU-level SARI surveillance **acceptable**?	25	1	1	27	0
Is the EU-level SARI surveillance system **timely** to meet its surveillance objectives?	16	8	3	27	0
Is the EU-level SARI surveillance system **representative** to meet its surveillance objectives?	14	6	5	25	2

EU: European Union; **SARI**: severe acute respiratory infection;

[a] Excluding the “No consensus” responses.
[b] When a country's responses are evenly split between "yes" and "no";

Supplementary Table S8 - Overview of questions, number of responses by objective/attribute, 27 European countries, 2022/23 (multiple-response-per-country approach)

**Questions**	**Yes**	**No**	**Unknown**	**Total**
Does the EU-level SARI surveillance system meet its **objective 1**: To monitor trends in severe respiratory infections and their impact on hospitalisations and in-hospital mortality?	39	4	7	50
Does the EU-level SARI surveillance system meet its **objective 2**: To ensure the early detection and response to unusual and unexpected events caused by common or emerging respiratory pathogens?	29	10	11	50
Does the EU-level SARI surveillance system meet its **objective 3:** To assess the impact of public health interventions on respiratory infections and inform disease preparedness?	24	8	18	50
Does the EU-level SARI surveillance system meet its **objective 4**: To identify risk factors for severe acute respiratory infection and death?	27	14	9	50
Does the EU-level SARI surveillance system meet its **objective 5**: To contribute to pathogen-specific SARI vaccine effectiveness monitoring?	28	9	13	50
Is the EU-level SARI surveillance **useful**?	45	1	4	50
Is the EU-level SARI surveillance **acceptable**?	45	1	4	50
Is the EU-level SARI surveillance system **timely** to meet its surveillance objectives?	28	13	9	50
Is the EU-level SARI surveillance system **representative** to meet its surveillance objectives?	25	11	14	50

EU: European Union; **SARI**: severe acute respiratory infection.

Supplementary Text S1 - Online questionnaire to assess surveillance attributes and identify SWOTs, 27 European countries, 2022/23

Introduction
This questionnaire is part of the SARI surveillance evaluation within the ECDC-funded projects to strengthen SARI surveillance. It is targeted to individuals responsible for SARI surveillance from your institution and gives you the opportunity to express your view on any required future system changes. Please note that your SARI national surveillance system is not being evaluated. This exercise is targeting the SARI surveillance at the EU-level. This questionnaire should take around 30 minutes to complete. The questionnaire responses will be treated as CONFIDENTIAL; the data will be analysed and only aggregated responses will be shown to ECDC in the form of a report. If you have any questions about this questionnaire or any aspect of the SARI surveillance evaluation, please do not hesitate to contact us. We thank you for your time in completing this important questionnaire and look forward to sharing the results with you.

Participant characterisation
- Country of your organisation
- Name of your organisation

Informed consent
After reading the information provided above about the SARI surveillance evaluation, please let us know if you are happy to provide informed consent for your answers to be analysed as part of this project and the aggregated results to be published in the project report and future scientific publication.

Do you provide informed consent? *Yes/No

Attribute - Meeting objectives

Definition: The extent to which the surveillance system has met its objectives.

Q1 - Does the EU-level SARI surveillance system meet its objective 1: To monitor trends in severe respiratory infections and their impact on hospitalisations and in-hospital mortality? *Yes/No/Unknown

Q1a - If No, please choose the THREE main reasons why not.

1. Lack of case-based data
2. Laboratory data not recorded in national databases (e.g. use of point of care tests in hospitals or patient self-tests in the community)
3. Insufficient capacity for virological subtyping/characterisation/sequencing
4. Difficulties collecting/linking data at national level (e.g. lack of unique identifier, data protection issues, low clinician motivation, lack of legal framework)
5. Difficulties sharing data to the European level (e.g. data protection issues)
6. Insufficient data completeness/validity on outcomes
7. Heterogeneity of SARI surveillance system between countries (e.g. different patient recruitment protocols, different case definitions, different testing strategies)
8. High workload or not enough resources

9. Lack of automation of systems

Q2 - Does the EU-level SARI surveillance system meet its objective 2: To ensure the early detection and response to unusual and unexpected events caused by common or emerging respiratory pathogens? *Yes/No/Unknown

Q2a - If No, please choose the THREE main reasons why not.

1. Lack of case-based data
2. Laboratory data not recorded in national databases (e.g. use of point of care tests in hospitals or patient self-tests in the community)
3. Insufficient capacity for virological subtyping/characterisation/sequencing
4. Difficulties collecting/linking data at national level (e.g. lack of unique identifier, data protection issues, low clinician motivation, lack of legal framework)
5. Difficulties sharing data to the European level (e.g. data protection issues)
6. Lack of comparable historical data (e.g. to ascertain what is unusual/unexpected)
7. Existing case definitions may miss unusual presentations or emerging respiratory pathogens
8. Insufficient flexibility (e.g. to detect unusual presentations or emerging respiratory pathogens)
9. Insufficient timeliness (e.g. to allow early detection)
10. Insufficient representativeness (e.g. to allow early detection)

Q3 - Does the EU-level SARI surveillance system meet its objective 3: To assess the IMPACT of public health interventions on respiratory infections and inform disease preparedness? *Yes/No/Unknown

Q3a - If No, please choose the THREE main reasons why not.

1. Lack of case-based data
2. Laboratory data not recorded in national databases (e.g. use of point of care tests in hospitals or patient self-tests in the community)
3. Insufficient capacity for virological subtyping/characterisation/sequencing
4. Difficulties collecting/linking data at national level (e.g. lack of unique identifier, data protection issues, low clinician motivation, lack of legal framework)
5. Difficulties sharing data to the European level (e.g. data protection issues)
6. Insufficient data completeness/validity on outcomes
7. Insufficient data completeness/validity on PH interventions (e.g. vaccination status, antiviral prophylaxis or treatment)
8. Heterogeneity of PH interventions between countries

Q4 - Does the EU-level SARI surveillance system meet its objective 4: To identify risk factors for severe acute respiratory infection and death? *Yes/No/Unknown

Q4a - If No, please choose the THREE main reasons why not.

1. Lack of case-based data
2. Laboratory data not recorded in national databases (e.g. use of point of care tests in hospitals or patient self-tests in the community)
3. Insufficient capacity for virological subtyping/characterisation/sequencing

4. Difficulties collecting/linking data at national level (e.g. lack of unique identifier, data protection issues, low clinician motivation, lack of legal framework)
5. Difficulties sharing data to the European level (e.g. data protection issues)
6. Insufficient data completeness/validity on outcomes
7. Insufficient data completeness/validity on risk factors
8. Lack of details on variables to collect risk factors (e.g. definitions of preconditions)

Q5 - Does the EU-level SARI surveillance system meet its objective 5: To contribute to pathogen-specific SARI vaccine effectiveness monitoring? *Yes/No/Unknown

Q5a - If No, please choose the THREE main reasons why not.

1. Lack of case-based data
2. Laboratory data not recorded in national databases (e.g. use of point of care tests in hospitals or patient self-tests in the community)
3. Insufficient capacity for virological subtyping/characterisation/sequencing
4. Difficulties collecting/linking data at national level (e.g. lack of unique identifier, data protection issues, low clinician motivation, lack of legal framework)
5. Difficulties sharing data to the European level (e.g. data protection issues)
6. Insufficient data completeness/validity on outcomes
7. Insufficient data completeness/validity on vaccination status
8. Heterogeneity of laboratory tests being used (e.g. PCR vs RADT)

Attribute - Usefulness

Definition: The ability of the surveillance system to provide data relevant for its intended use.

Q6 - Is the EU-level SARI surveillance useful? *Yes/No/Unknown

Q6a - If No, please choose the THREE main reasons why it is NOT useful.

1. Does not meet any of the surveillance objectives
2. Lack of case-based data
3. Lack of comparability between hospitals/countries (different case definitions, methodologies)
4. SARI surveillance outputs are not easily available/not well known
5. SARI surveillance data are not fully used
6. Insufficient representativeness (e.g. results may reflect what is happening in larger countries)
7. Insufficient acceptability (e.g. to data are not collected/shared due to lack of clinician motivation, increased workload or public health importance of SARI surveillance not being recognised)

Attribute - Acceptability

Definition: The willingness to participate in the SARI surveillance system at EU-level and to provide accurate, consistent, complete and timely data.

Q7 - Is the EU-level SARI surveillance acceptable? *Yes/No/Unknown

Q7a - If No, please choose the THREE main reasons why it is NOT acceptable.

1. The public health importance of SARI surveillance is NOT recognised at hospital level
2. The public health importance of SARI surveillance is NOT recognised at national level
3. Increased workload for hospital staff / conflicting priorities
4. Ethical issues to collect/share data (e.g. SARI surveillance considered as research, requirements for informed consent)
5. Data protection to collect/share data (e.g. data linkage, data sharing at EU level, risk of patient identification due to small counts)
6. Lack of resources
7. Weekly data submission is challenging
8. Lack of automation in data collection process

Attribute - Timeliness

Definition: Timeliness is the time interval between steps of the data flow in a surveillance system (e.g. timeliness of inputs: the time between the diagnosis of a case and the reporting of this case at EU level; timeliness of outputs: the time between the reporting of cases at EU level and the production of outputs).

Q8 - Is the EU-level SARI surveillance system timely to meet its surveillance objectives? *Yes/No/Unknown

Q8a - If No, please choose the THREE main reasons why it is NOT timely.

1. Case-based data collection and submission is time consuming
2. Manual data extraction and submission is slow/inefficient
3. Delays in laboratory testing
4. High workload or insufficient resources to collect/submit data (especially during surge activity periods)
5. Requirement to wait for discharge to submit data (e.g. if submission depends on discharge ICD-10 codes or discharge outcomes)
6. High number of variables to be collected

Attribute - Representativeness

Definition: The data from the EU-level SARI surveillance system should be representative of the SARI cases occurred in the population of the EU/EEA, namely their distribution in terms of geography (regions, countries), demographics (age group, sex, risk groups) and severity (hospitalised, ICU and deaths).

Q9 - Is the EU-level SARI surveillance system representative to meet its surveillance objectives? *Yes/No/Unknown

Q9a - If No, please choose the THREE main reasons why it is NOT representative.

1. Insufficient age group representativeness (e.g. focus is on adult age groups)
2. Insufficient urban/rural representativeness (e.g. focus is on urban areas)

3. Insufficient geographical representativeness (e.g. poor coverage of participating countries)
4. Insufficient socio-economic group or private/public hospital representativeness
5. Insufficient representativeness of severity (e.g. the system focuses on more severe hospitalisations, i.e. ICU admissions)
6. Insufficient representativeness of mortality (e.g. the system misses those who died within 24h of admission or after discharge)
7. Lack of harmonised SARI patient recruitment protocols
8. Lack of harmonised SARI case definitions
9. Data collection and sharing is limited by ethical and data protection issues

SWOT analysis - Strengths

Definition: Strengths are internal factors, within an organisation control, that can enable, create and sustain success. For example: What are the greatest strengths? What do we do well? How is SARI surveillance different from other surveillance systems? What is unique about SARI surveillance?

Q10 - Reflecting on the Strengths of the SARI surveillance system, please select the THREE you believe are the most important.

1. Timeliness of the system
2. Representativeness of the system (e.g. multi-country system, large sample size)
3. Integrated surveillance with laboratory component for several respiratory pathogens
4. Standard protocol, objectives and case definitions
5. Systematic way of identifying severe patients for being sampled and monitored
6. Covers multiple objectives (monitoring of hospitalisation trends, severity, burden of disease, early warning and preparedness, impact of PH interventions, e.g. vaccine effectiveness and vaccination
7. Being part of an experienced European network (collaboration, learning from each other, highly motivating)

SWOT analysis - Weaknesses

Definition: Weaknesses are internal factors, within an organisation control, that can create vulnerability or prevent achieving objectives. For example: What are the greatest weaknesses? What makes SARI surveillance vulnerable to bias? What prevents SARI surveillance from achieving its objectives? What complains do we frequently hear about SARI surveillance? What are the biggest current obstacles? What advantages other systems have that SARI surveillance doesn't?

Q11 - Reflecting on the Weaknesses of the SARI surveillance system, please select the THREE you believe are the most important.

1. Heterogeneity of SARI surveillance system between countries (e.g. different patient recruitment protocols, different case definitions, different testing strategies)
2. Complex system with many stakeholders making it difficult to implement and sustain
3. Data protection issues at national level, e.g. to collect case-based data and do data linkages
4. Data protection issues to share data internationally
5. SARI surveillance reporting is not mandatory

6. SARI surveillance is not a public health priority at national level
7. Lack of sustainable funding to collect data, do analysis and produce outputs
8. Resource intensive for hospitals and national public health institutes
9. There is lack of interest in surveillance at the hospital level and is difficult to keep them motivated
10. Difficult to maintain the system in periods of surge activity when hospitals and clinicians are very busy
11. Limited capacity at country level for automation and data linkage (e.g. unavailability or difficulties linking vaccination data)

SWOT analysis - Opportunities

Definition: Opportunities are external factors, uncontrollable, that can make surveillance more efficient. For example: Are there any changes in regulations that might benefit SARI surveillance? Are there new technologies that may improve SARI surveillance attributes? Are there new methods/processes that may help to reduce costs or burden in data collection/analysis? Are there any changes in surveillance systems that might benefit SARI surveillance?

Q12 - Reflecting on the Opportunities of the SARI surveillance system, please select the THREE you believe are the most important.

1. Developments of new methods to collect data, e.g. artificial intelligence for text mining
2. Improvements in IT infrastructures in hospitals/laboratories to allow data linkage and data sharing
3. Financial and technical support from ECDC-funded projects
4. Increased political and scientific support due to the COVID-19 pandemic
5. Transition from universal surveillance to sentinel integrated surveillance e.g. fear of loss of data from policy makers
6. Inclusion of SARI as a notifiable disease at EU level in 2024
7. New EU regulation on serious cross-border threats (2022/2371)
8. Initiatives at EU level that can benefit SARI surveillance: United4Surveillance, Direct Grants, European Health Data Space
9. New RSV vaccines may increase public and authorities' interest in SARI surveillance

SWOT analysis - Threats

Definition: Threats are external factors, uncontrollable, that can negatively impact surveillance. For example: Any changes in policy that can negatively impact SARI surveillance? Are there pressures in the hospital setting, or public health institutes that could negatively impact the performance of SARI surveillance? Are there other priorities in funding? Are there alternative surveillance systems being developed? Are there alternative projects occurring that could negatively impact SARI surveillance?

Q13 - Reflecting on the Threats to the SARI surveillance system, please select the THREE you believe are the most important.

1. Lack of sustainable funding
2. Lack of transparency and clarity on the use of SARI surveillance at EU level by third parties

3. Systems implemented during the pandemic may cease to exist when COVID-19 is not considered a public health emergency of international concern (PHEIC)
4. End of mandatory testing for SARS-CoV-2
5. Changes in policy priorities could disrupt SARI surveillance and lead to resource re-allocation
6. Lack of legal framework for reporting and linking SARI surveillance data at national level
7. Hospitals under pressure during surge periods
8. Overlap of projects on an EU level may lead to confusion
9. Duplication of activities across surveillance systems (i.e. COVID-19, Influenza, SARI)
10. Laboratory data not recorded in national databases (e.g. use of point of care tests in hospitals or patient self-tests in the community)
11. Loss of data due to overloading of the hospitals (i. e. during pandemic) or in case of failure of IT systems
12. War, humanitarian emergencies/disasters

Additional comments

Please add any comments or feedback about this questionnaire or the SARI surveillance evaluation.

Conclusion

We thank you for your time in completing this important questionnaire.
